# The effect of a traditional Chinese quadri-combination therapy and its component quercetin on recurrent spontaneous abortion: A clinical trial, network pharmacology and experiments-based study

**DOI:** 10.3389/fphar.2022.965694

**Published:** 2022-10-19

**Authors:** Jing Zhou, Lisha Li, Xinyao Pan, Jing Wang, Qing Qi, Hongmei Sun, Chuyu Li, Ling Wang

**Affiliations:** ^1^ Laboratory for Reproductive Immunology, Obstetrics and Gynecology Hospital of Fudan University, Shanghai, China; ^2^ The Academy of Integrative Medicine of Fudan University, Shanghai, China; ^3^ Shanghai Key Laboratory of Female Reproductive Endocrine-Related Diseases, Shanghai, China

**Keywords:** quadri-combination therapy, recurrent spontaneous abortion, quercetin, mitochondrial dynamics, DRP1, miR-34a-5p

## Abstract

**Objective:** To explore the effect and mechanisms of a traditional Chinese quadri-combination therapy [Bushen, Yiqi, Lixue and Yangtai (BYLY)] in treating recurrent spontaneous abortion (RSA).

**Methods:** A clinical trial was conducted to study the effect of BYLY on RSA. Pharmacological network analysis and UPLC-Q/TOF-mass spectrometry (MS) were applied to investigate the key active component in BYLY and potential targets. Cellular experiments based on former results were performed to examine the mechanism of BYLY in the treatment of RSA.

**Results:** Four hundred and eighty participants enrolled in the clinical trial. The results showed that, compared with the use of BYLY or duphaston alone, a combination of duphaston and BYLY could decrease the early abortion rate in RSA (*p* < 0.001). Network pharmacological analysis indicated that BYLY contained 132 active components and 146 core targets, and the quercetin maybe the key effective component. *In vitro* experiments found that pretreatment of quercetin at the correct concentration (2 μM) prevented hypoxia-induced viability and proliferation reduction, and apoptosis and mitochondrial dysfunction. Furthermore, quercetin could modulate mitochondrial fission/fusion balance in trophoblasts, and specifically decrease the expression of Drp1 by regulating miR-34a-5p.

**Conclusion:** BYLY could improve pregnancy outcomes of RSA, based on multi-components and multi-targets. The protective effect of quercetin on trophoblasts, through decreasing Drp1 expression via regulating miR-34a-5p, might be one possible effective mechanism.

## Graphical Abstract

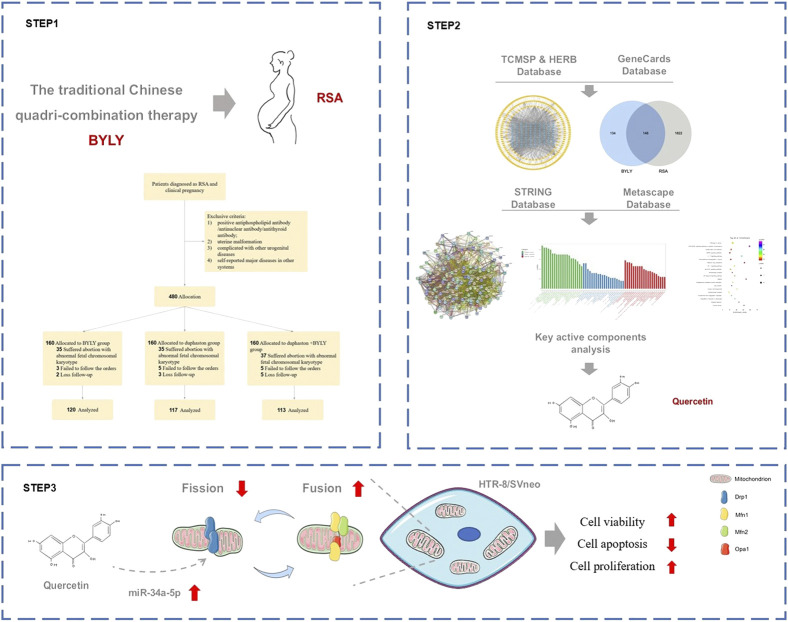

## 1 Introduction

Recurrent spontaneous abortion (RSA), which affects ∼2.5% of women, involves a failure of two or more pregnancies before 20–24 weeks of gestation with the same sexual partner (Davies et al., 2016; Dimitriadis et al., 2020). Since more than half of RSA remains unexplained, it is difficult to make a breakthrough in RSA therapy. Clinically, progestin supplement and immunotherapy are the main treatments for RSA. Progesterone and progestogen are often used in the first trimester to induce secretory endometrium to make it suitable for implantation. Progesterone or progestogen can reduce miscarriage rate in women, but have little effect on live birth rate (Coomarasamy et al., 2015; Saccone et al., 2017; Ismail et al., 2018). Furthermore, for RSA patients with no diagnosis of inadequate secretion of progesterone, the effectiveness of progesterone supplementation is questioned (Haas et al., 2019). Since the development of immunotherapy, weak evidence shows the increased live birth rate after treatment with lymphocyte immunotherapy, intravenous immunoglobulin and cytokine growth factor treatment (Group, 1994; Christiansen et al., 2002; Scarpellini and Sbracia, 2009). Both larger sample sizes and high-quality trials are needed for immunomodulatory therapy; however, the risks associated with blood products and the high price also limit the use of immunotherapy. During recent decades, various supplementary strategies have been studied to improve the outcomes of RSA, such as antiplatelet therapy, docosahexaenoic acid (DHA), and folic acid (Rogers et al., 2013; Hua et al., 2016; Nana et al., 2021). Traditional Chinese Medicine (TCM) has a long history and has considerable therapeutic effect in the prevention and treatment of several diseases (Lin et al., 2017; Qi and Tang, 2021; Zhang et al., 2021). The full effects of TCM and its underlying mechanism on RSA are require further exploration.

For patients with RSA, growing evidence shows that the intervention of TCM might increase pregnancy success rate without obvious adverse events (Li et al., 2020; Wang P. et al., 2021). In TCM, RSA refers to “Hua tai”, a Chinese term, which means the womb cannot function well to support fetal development and the fetus has difficulty adhering to the womb (implanting). A healthy pregnancy in TCM requires sufficient “Qi” and “Xue” (“blood”) supply, both of which rely on the function of “Shen” (“kidney”). Thus, “Bushen” (tonifying kidney) is the key principle in treating RSA and is commonly combined with “Yiqi” (reinforcing kidney-Qi) or “Lixue” (preventing blood stasis). “Bushen-Yiqi-Lixue-Yangtai” (BYLY) compounds are innovative TCM that have been used in the prevention and treatment of RSA. Compared with other TCM for abortion prevention, BYLY simultaneously tonifies “Shen”, “Qi”, and “Xue”, in addition to nourishing the fetus. However, due to its various components and complex interactions between herbs, the underlying mechanism of this quadri-combination therapy remain unexplained.

First, a preliminary clinical trial was conducted to observe the effect of BYLY in treating RSA. Then network pharmacology analysis and UPLC-Q/TOF-mass spectrometry (MS) were performed to explore the active components and the underlying mechanisms. Furthermore, cellular experiments based on former results were used to demonstrate the potential mechanisms of BYLY.

## 2 Materials and methods

### 2.1 A preliminary clinical trial

#### 2.1.1 Trial oversight

**FIGURE 1:**
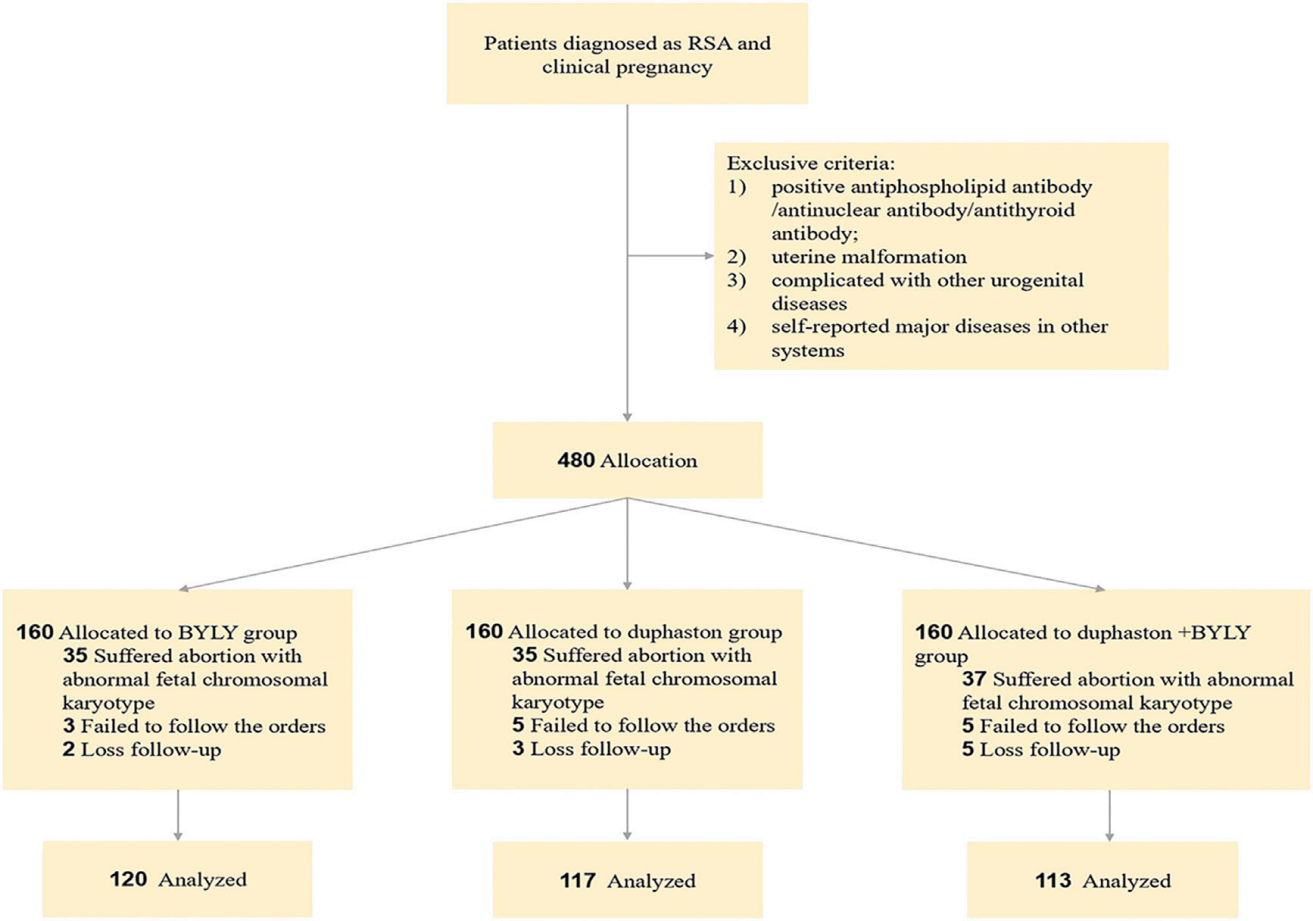
Flowchart of study on BYLY for patient with RSA. Abbreviation: BYLY, *Bushen Yiqi Lixue Yangtai* compounds; RSA, recurrent spontaneous abortion.

This prospective clinical trial was conducted in the Obstetrics and Gynecology Hospital of Fudan University from 1 September 2018, through 30 December 2020 and was approved by the hospital’s Ethics Committee (No. 2019–57). The flow of patients through the study is shown in Figure 1.

#### 2.1.2 Patient recruitment

RSA patients diagnosed with clinical pregnancy were enrolled in this trial. RSA diagnosis was based on the European Society for Human Reproduction and Embryology (ESHRE) Guideline (Davies et al., 2016) and clinical pregnancy diagnosis was based on β-human chorionic gonadotrophin (β-HCG) level. RSA patients were further selected based on the following inclusive criteria: 1) signing an informed consent; 2) 20–40 years old; 3) <6 gestational weeks; 4) regular menstrual cycle; 5) hormone levels within normal range; 6) negative antiphospholipid antibody/antinuclear antibody/antithyroid antibody; 7) fallopian tube and uterine cavity with normal morphology; 8) whose partner had healthy semen; 9) chromosomal karyotype of couples were normal. Exclusion criteria included: 1) complicated with other urogenital diseases; 2) self-reported major diseases in other systems. The medication and follow-up would cease if patients 1) asked for termination; 2) failed to follow the orders or failed to be followed up; 3) suffered abortion with abnormal fetal chromosomal karyotype; 4) were diagnosed with an ectopic pregnancy.

#### 2.1.3 Design and treatment

In total, 480 patients were recruited and assigned to the BYLY group, duphaston group, and duphaston + BYLY group in a 1:1:1 ratio according to patient preference and clinician judgement. In the BYLY group, patients received BYLY treatment (Table 1) (one compound, once daily) available from the pharmacy of the Obstetrics and Gynecology Hospital of Fudan University (Patent Application Number: 202210037393.8); in the duphaston group, they received duphaston treatment (10 mg, Abbott Biologicals B.V) (10 mg, three times daily); and in the duphaston + BYLY group they received both BYLY and duphaston as described above. The total treatment period was 14–28 days, adjustments being made by clinicians according to the condition of each patient. The treatment would cease when β-HCG > 100,000, or fetal cardiac activity was detected under ultrasound.

**TABLE 1:** Composition of BYLY.

Herb	Latin name
*Baishao*	*Paeonia lactiflora Pall*
*Baizhu*	*Atractylodes macrocephala Koidz*
*Chuanxiong*	*Ligusticum striatum*
*Danggui*	*Angelica sinensis*
*Dangshen*	*Codonopsis pilosulae*
*Duzhong*	*Eucommia ulmoides*
*Huangqi*	*Astragalus mongholicus*
*Huangqin*	*Scutellaria baicalensis*
*Nanguadi*	*Cucurbita moschata*
*Sangjisheng*	*Taxillus chinensis*
*Shengdihuang*	*Rehmannia glutinosa*
*Tusizi*	*Cuscuta chinensis*
*Xuduan*	*Dipsacus asper*
*Zhumagen*	*Boehmeria nivea*
*Zisugeng*	*Perilla frutescens* var. *crispa*

#### 2.1.4 Outcomes

The primary outcome of this study was early spontaneous abortion, defined as pregnancy loss before 12 weeks of gestation. Tests including blood routine, liver and kidney function as well as reproductive hormone levels were conducted to assess the safety of the procedures. Patients were followed up until the end of the pregnancy.

### 2.2 Pharmacological network analysis and UPLC-Q/TOF-MS

#### 2.2.1 Prediction and analysis of active components and its targets in BYLY

With the standards of oral bioavailability (OB) ≥30% and the drug-like (DL) ≥0.18, active components of BYLY were filtered from the Traditional Chinese Medicine Systems Pharmacology Database and Analysis Platform (TCMSP, https://www.tcmsp-e.com) and HERB (http://herb.ac.cn). Putative targets of active compounds were collected from TCMSP. The data of active components and their target genes were imported in Cytoscape 3.8.2 software to build a components-targets network.

#### 2.2.2 Collection of RSA-related targets and selection of core regulatory genes

Since all putative targets of active components obtained from TCMSP were protein names, all protein names were coverted to gene names by the UniProt database (https://www.uniprot.org). RSA-related genes were obtained by searching “recurrent spontaneous abortion” on GeneCards (https://www.genecards.org). Putative gene targets of active components and RSA-related genes were compared to find the overlapped gene targets, which were considered to be the core regulatory genes of BYLY regulation on RSA.

#### 2.2.3 Protein-protein interaction network and crucial subnetwork

A PPI network of core regulatory targets was conducted using the STRING database (https://string-db.org). The species was *“Homo sapien”* and confidence was 0.4. The PPI network was then imported into Cytoscape 3.8.2 software to retrieve the crucial subnetwork. The Cytohubba and CytoNCA plugin was used to select the top 20 nodes and reconstruct the crucial network.

#### 2.2.4 GO enrichment analysis and KEGG pathway enrichment analysis

GO enrichment analysis and KEGG enrichment analysis were conducted to analyze the functions of these core regulatory genes using the Metascape database (https://metascape.org/) The results underwent visualization conversion using https://cloud.oebiotech.cn/.

#### 2.2.5 UPLC-Q/TOF-MS

The BYLY solution contains 15 Chinese herbs (Table 1) and was obtained from the pharmacy of the Obstetrics and Gynecology Hospital of Fudan University (Patent Application Number: 202210037393.8). Briefly, all herb powders were dissolved in water in specific proportions and stored at 4°C. Quercetin (analytical standard, ≥98.5%) was purchased from Aladdin Biochemical Technology Co., Ltd. Each BYLY sample was diluted with a 1-fold volume of methanol and quercetin was dissolved to 10 μg/ml with methanol. All samples were vortexed and centrifuged at 15 000 rpm for 5 min.

Chromatographic analysis was performed on a Waters ACQUITY UPLC System (Waters Corporation, United States). Column temperature was 45°C and ultraviolet wavelength was 200–600 nm. The injection volume was set as 1 μl. The mobile phase was composed of phase A: 5 mM amine acetate +0.1% formic acid (in water) and phase B: 0.1% formic acid (in acetonitrile/methanol = 9/1, v/v). Gradient elution at a speed of 0.4 ml/min was initiated from 5%B for 1 min, then increased linearly to 70%B over a period of 23 min, then increased linearly to 95% B within 2 min and maintained for 2 min before a final decrease to 5%B to equilibrate the column.

The MS experiment was conducted by Synapt G2-Si MS. The ionization mode involved negative electrospray. The source temperature and the desolvation gas temperature were set as 120 and 350°C, respectively. The capillary voltage and the cone voltage were set as 3.0 kV and 40 V, respectively. The desolvation gas was nitrogen and the collision gas was argon. Leucine Enkphalin was used as the lock mass (*m/z* 554.2615).

Data ranging from 50 to 1200 Da were obtained in two independent acquisition functions with rapid switching between them in 20 ms. The first acquisition function had lower collision energy, with settings of 2 eV for trap collision energy and 2 eV for transfer collision energy, which collected unfragmented data with low energy. The second one was set as 4–18 eV for trap collision energy and 10–35 eV for transfer collision energy.

Data acquisition was performed with Masslynx V4.1. Data processing was carried out by Metabolynx. Structural elucidation was performed by MassFragmentTM.

### 2.3 Cell experiments

#### 2.3.1 Cell culture and treatment

The HTR-8/SVneo cell line was obtained and conserved at the Institution of Obstetrics and Gynecology Hospital of Fudan University (Shanghai, China). Cells were cultured in RPMI 1640 Medium (Gibco; Thermo Fisher Scientific, Inc.) supplemented with 10% (v/v) fetal bovine serum (FBS) (Gibco; Thermo Fisher Scientific, Inc.) and 1% (v/v) penicillin-streptomycin solution (New Cell and Molecular Biotech Co., Ltd.) at 37°C in a 5% CO_2_ humidified atmosphere. Quercetin (Q4951, Sigma-Aldrich Inc.) powder was dissolved with dimethyl sulfoxide (DMSO) (Sango Biotech Co., Ltd.) to 200 mg/ml for storage and diluted with culture medium to 2, 5, and 10 μM for cell treatments. The concentration of DMSO in the final culture medium was less than 0.01% (v/v). For hypoxia incubation, 70% confluency of HTR-8/SVneo cells were pretreated with a dose dependent quercetin (0/2/5/10 μM) for 48 h and were transferred to a hypoxia incubator chamber (MIC101, Billups-rothenberg) with 1% O_2_, 5% CO_2_, and an N_2_ balanced atmosphere for 48 h. For normoxic incubation, cells were cultured with complete medium for 96 h.

#### 2.3.2 Cell viability assay

Cell viability was evaluated using a cell counting kit-8 (CCK-8) (Yeasen Biotechnology Co., Ltd.). HTR-8/SVneo cells were cultured in 96-well plates with 6 × 10^3^ cells and 200 μL per well. After treatment, 20 μl CCK-8 were added into each well and cultured in the dark for 2 h. Absorbance at 450 nm of each well was measured using a microplate reader. The results were normalized to the hypoxia group without quercetin treatment.

#### 2.3.3 Cell apoptosis assay

Cell apoptosis was performed by labeling cells with Annexin V and propidium iodide (PI) (Biolegend Inc.). In brief, 6 × 10^3^ treated cells were washed twice with phosphate-buffered saline (PBS) and resuspended with binding buffer (Biolegend Inc.). After adding 5 μl Annexin V for 10 min and 3 μl PI for 5 min, cellular apoptosis rate was quantified by flow cytometry (CytoFLEX; Beckman Coulter, Inc.). Cells in the Annexin V +/PI + field and Annexin V +/PI - field were regarded as apoptosis events. Data were analyzed using Flowjo 10.4.0 software.

#### 2.3.4 Cell proliferation assay

Cell proliferation detection was performed using a Cell-Light EdU Kit (RiboBio Co., Ltd.) according to the manufacturer’s instructions. Edu-positive cells were scanned and calculated using fluorescence microscopy (Nikon, Japan) from at least three random fields.

#### 2.3.5 Mitochondrial membrane potential measurement

A JC-1 assay kit (Yeasen Biotechnology Co., Ltd.) was purchased to assess MMP. In total, 1 × 10^4^ treated cells in each group were stained with JC-1 following the manufacturer’s protocol. The fluorescence intensity of JC-1 aggregates (red fluorescence)/monomers (green fluorescence) were detected by flow cytometry (CytoFLEX; Beckman Coulter, Inc.). Data were analyzed using Flowjo 10.4.0 software.

#### 2.3.6 Observation of mitochondria morphology

Cells were fixed with fixative for transmission electron microscope (TEM) (Servicebio Technology Co., Ltd.). Briefly, samples were prepared through post-fix with 1% OsO_4_, dehydrated, embedded, underwent polymerization, and ultrathin sectioning according to the TEM staining protocol provided by Servicebio. Samples were observed under TEM (HT7700; HITACHI, Japan) and images were taken. At least three random fields of each sample were observed.

#### 2.3.7 Bioinformatics analysis and dual-luciferase report assay

The upstream miRNAs were obtained from starBase (https://starbase.sysu.edu.cn), miRDB (http://mirdb.org), TargetScan (https://www.targetscan.org), and miRWalk (http://mirwalk.umm.uni-heidelberg.de). Gene target was set as DNM1L and species was human. The binding sites of miR-34a-5p on wild-type (wt) sequence of Drp1 were muted to generate a mutant (mut) sequence of Drp1. The Drp1-wt and Drp1-mut sequences were cloned to pSI-Check2 vectors, generating Drp1-3′UTR-wt and Drp1-3′UTR-mut, respectively (Hanbio Biotechnology Co., Ltd.). Drp1-3′UTR-wt or Drp1-3′UTR-mut luciferase report vectors and miR-34a-5p mimics or NC mimics were co-transfected into 293 T cells. Luciferase activity was analyzed by the dual-Luciferase system (Promega Biotech Co., Ltd.).

#### 2.3.8 Cell transfection

The NC-agomir, miR-34a-5 p agomir, NC-antagomir, and miR-34a-5 p antagomir were purchased from GenePharma Co.,Ltd. HTR-8/Svneo cells were transfected with 1 μg vectors with lipofectamine 3,000 (Thermo Fisher Scientific Inc.) following the manufacturer’s instructions.

#### 2.3.9 Quantitative reverse transcription polymerase chain reaction (qRT-PCR)

RNA from treated cells was extracted using RNAiso Plus (Takara Biomedical Technology (Beijing) Co., Ltd.). RNA concentration quantification was performed by NanoDrop one (Thermo Fisher Scientific Inc.). Extraction of cDNA was obtained using PrimeScript™ RT reagent Kit (Perfect Real Time) (Takara) and Veriti 96 well thermal cycler (Thermo Fisher Scientific Inc.) following the manufacturer’s instructions. For miRNAs expression detection, miRNA cDNA Synthesis Mix and miRNA qPCR Mix (WcGene Biotech Technology Ltd.) were used for reverse transcription and PCR. PCR was conducted by Quantstudio six Flex (Thermo Fisher Scientific Inc.) to evaluate the expression level of mRNA and miRNA. β-actin and U6 acted as the loading control. The primers designed are shown in Supplementary Table S4. The universal reverse primer for miRNAs PCR was delivered by a miRNA qPCR Mix kit.

### 2.4 Statistical analysis

For clinical trials, continuous variables were analyzed in normal distribution by the Kolmogorov-Smirnov test. Data were expressed as mean ± standard deviation (‾x ± SD) and analyzed by one-way analysis of variance (ANOVA) for normal distributions, and were expressed as median (interquartile range) and analyzed by Mann-Whitney test if not. Categorical variables were expressed as percentages and analyzed by Pearson χ^2^ test. Data analysis was conducted using SPSS v.26. For cell experiments, all data values of at least three independent experiments were represented as ‾x ± SD. The Shapiro-Wilk test was employed for normal distribution. Data were analyzed with Student’s *t-*test and ANOVA by GraphPad Prism v.8. Asterisks indicate statistical significance of corresponding comparisons.

## 3 Results

### 3.1 BYLY decreased early abortion rate of RSA patients

Between 1 September 2018, and 30 December 2020, a total of 480 RSA outpatients met the inclusive and exclusive criteria and were enrolled into the trial. In total, 130 patients failed to finish the medication and follow-up (40 in the BYLY group, 43 in the duphaston group, 47 in the duphaston + BYLY group) (Figure 1). The baseline characteristics including age and abortion times of each group were comparable (Table 2). The early abortion rate was 10.62% (12/113) in the duphaston + BYLY group, 29.17% (35/120) in the BYLY group, and 29.06% (34/117) in the duphaston group **(** Table 2 **)**. Compared with the BYLY and duphaston groups, the combination of duphaston and BYLY therapy showed a decreased early abortion rate (*p* < 0.001).

**TABLE 2:** Baseline characteristics and pregnancy outcomes in study groups.

	BYLY(*N* = 120)	Duphaston(*N* = 117)	Duphaston + BYLY(*N* = 113)
Age, median (IQR), y	29 (27.25–32.00)	30 (28–32)	31 (28–33)
Number of abortion, mean (IQR)	2 (2–3)	2 (2–3)	2 (2–3)
Early abortion rate, No. (%)	35 (29.17%)	34 (29.06%)	12 (10.62%) *

Abbreviation: BYLY, Bushen Yiqi Lixue Yangtai; IQR, interquartile range.

^a^ Duphaston + BYLY, vs BYLY, *p* < 0.001; Duphaston + BYLY, vs Duphaston, *p* < 0.001.

### 3.2 Acquisition of active compounds and its targets in BYLY

**FIGURE 2:**
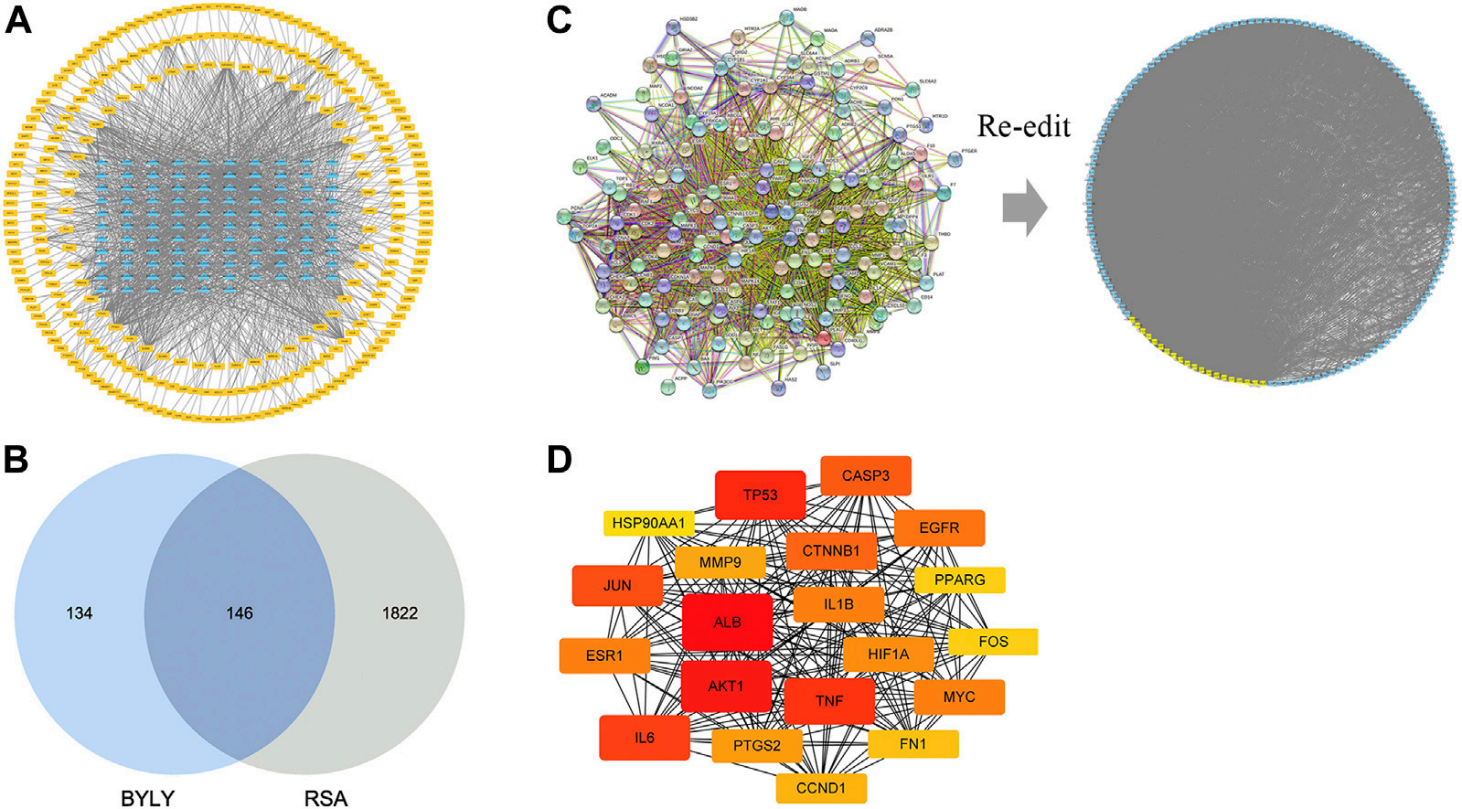
Components and targets analysis of BYLY in treating RSA. **(A)** Components-targets network. Blue triangles represented components. Yellow rectangles represented targets. **(B)** The core regulatory genes of BYLY in treating RSA. **(C)** PPI network of core regulatory targets from STRING and Cytoscape software. Yellow rectangles represented the top 20 target ranked by degree centrality with a descending order. Blue rectangles represented other targets. **(D)** PPI network of the top 20 targets. Rounded rectangles represented targets and the one with larger height or darker color represented greater degree centrality. Abbreviation: BYLY, *Bushen Yiqi Lixue Yangtai* compounds; RSA, recurrent spontaneous abortion.

Our preliminary clinical trial demonstrated the effect of BYLY in RSA therapy to some extent. To uncover the mechanisms of BYLY, network pharmacological methodology was applied. After filtering with the standard OB ≥ 30% and DL ≥ 0.18, 132 active compounds were retrieved from the 15 herbs in BYLY (Supplementary Table S1). Fifteen active compounds were derived from more than one herb in BYLY and three active compounds, beta-sitosterol, sitosterol, and quercetin, were present in more than five herbs in BYLY (Supplementary Table S2). Collecting all disease targets of these 132 active compounds, a components-targets network was constructed (Figure 2A). Yellow rectangles refer to targets and blue triangles represent components. Ranking the active compounds by degree in a descending order, quercetin, kaempferol, luteolin, wogonin, and 7-O-methylisomucronulatol were the top five molecules (Table 3). Quercetin had a degree of 141, that refers to 141 targets, showing its crucial role in BYLY.

**TABLE 3:** The crucial active components in BYLY.

Molecular id	Molecular name	Degree	Counts of core regulatory targets	Oral bioavailability (OB)%	Drug-like (DL)
MOL000098	Quercetin	141	96	46.43	0.28
MOL000422	Kaempferol	59	39	41.88	0.24
MOL000006	Luteolin	55	44	36.16	0.25
MOL000173	Wogonin	45	32	30.68	0.23
MOL000378	7-O-methylisomucronulatol	44	25	74.69	0.30

### 3.3 PPI network analysis of core targets

In total, 1968 targets of RSA and 280 targets of active ingredients from BYLY were retrieved from the databases and compared. Of these, 146 overlapping genes were considered as the core targets of BYLY in RSA management (Figure 2B and Supplementary Table S3). These 146 genes were imported into STRING to obtain a PPI network which contained 146 nodes (proteins) and 3048 edges (interactions) (Figure 2C). Further analysis of the PPI network took place with Cytoscape software with six parameters of nodes: betweenness centrality (BC), closeness centrality, degree centrality (DC), eigenvector centrality (EC), network centrality (NC), and local average connectivity (LAC), and 20 crucial proteins were selected and ranked with DC. Each rounded rectangle node represents a gene and those with a greater height or darker color represent a greater degree, which should be given more attention (Figure 2C). The top 20 nodes with higher degree were *ALB, AKT1, TP53, TNF, IL6, JUN, CASP3, CTNNB1, EGFR, ESR1, MYC, IL1B, HIF1A, PTGS2, MMP9, CCND1, FN1, PPARG, FOS,* and *HSP90AA1* (Figure 2D and Table 4).

**TABLE 4:** The top 20 core regulatory genes.

Gene name	Protein name	Degree
*ALB*	Albumin	228
*AKT1*	RAC-alpha serine/threonine-protein kinase	216
*TP53*	Cellular tumor antigen p53	210
*TNF*	Tumor necrosis factor	204
*IL6*	Interleukin-6	202
*JUN*	Transcription factor AP-1	192
*CASP3*	Caspase-3	190
*CTNNB1*	Catenin beta-1	184
*EGFR*	Epidermal growth factor receptor	182
*ESR1*	Estrogen receptor	180
*MYC*	Myc proto-oncogene protein	180
*IL1B*	Interleukin-1 beta	180
*HIF1A*	Hypoxia-inducible factor 1-alpha	178
*PTGS2*	Prostaglandin G/H synthase 2	176
*MMP9*	Matrix metalloproteinase-9	172
*CCND1*	G1/S-specific cyclin-D1	168
*FN1*	Fibronectin	164
*PPARG*	Peroxisome proliferator-activated receptor gamma	160
*FOS*	Proto-oncogene c-Fos	160
*HSP90AA1*	Heat shock protein HSP 90-alpha	158

### 3.4 GO enrichment analysis and KEGG pathway enrichment analysis

**FIGURE 3:**
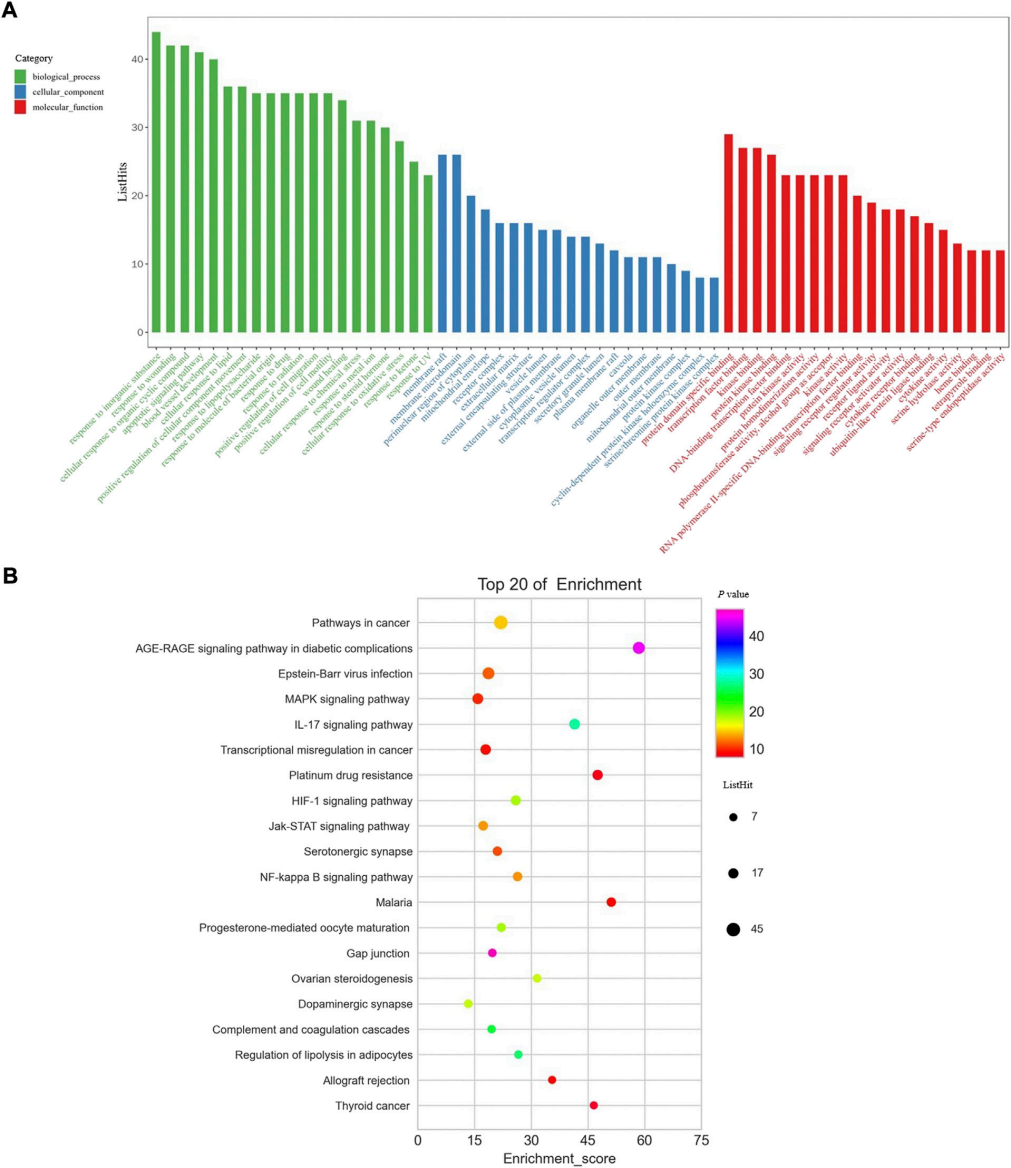
GO enrichment analysis and KEGG pathway enrichment analysis. **(A)** GO enrichment analysis. Green columns represent BP; blue columns represent CC and red columns represent MF. **(B)** KEGG pathway enrichment analysis. Abbreviation: BP, biological process; CC, cellular component; MF, molecular function.

To systematically explore the biological function, GO enrichment and KEGG pathway enrichment analysis were performed for these 146 core targets. GO enrichment analysis uncovered the function of genes by biological process (BP) (green bars), cellular component (CC) (blue bars), and molecular function (MF) (red bars). The top 20 enrichment data in BP, CC, and MF were retrieved respectively (Figure 3A). Response to inorganic substance, response to wounding, cellular response to organic cyclic compound, apoptotic signaling pathway, and blood vessel development were the most significant BP that core targets from BYLY may involve. Membrane raft and membrane microdomain were the main CP; and protein domain specific binding, transcription factor binding, kinase binding, and protein kinase binding were the key MF. KEGG pathway enrichment analysis identified proper pathways and the core targets might be involved. The top 20 significant pathways were visualized in a bubble diagram (Figure 3B). Enrichment score in each pathway indicated the ratio of differentially expressed genes to all genes in this pathway. The size of bubbles stood for the differentially expressed gene counts.

### 3.5 Quercetin plays a crucial role in treating RSA

A component with more targets was considered to take a more important role in BYLY. Thus, targets of quercetin, kaempferol, luteolin, wogonin, and 7-O-methylisomucronulatol were Table 3 compared with the 146 core targets to evaluate their role in BYLY when treating RSA. Respectively, 96 targets putatively showed effects among the 141 targets of quercetin; 39 targets among the 59 targets of kaempferol; 44 targets among the 55 targets of luteolin; 32 targets among the 45 targets of wogonin; and 25 targets among the 44 targets of 7-O-methylisomucronulatol (Table 3). The importance of those overlapping targets was then analyzed with each other and with the top 20 crucial nodes in PPI analysis (Supplementary Figure S2 and Table 5). Targets identified in the top 20 targets are underlined and overstruck. Diagrams in the top row show the area which genes in the corresponding rank came from. Six reduplicative genes were contained in all sections, but only three genes were identified from the top 20 genes. However, among the 36 exclusive genes from quercetin, *FOS, HIF1A, IL1B,* and *MYC* were also in the top 20 genes. One reduplicative gene, *FN1*, was found in particular targets of wogonin. Taken together, these results further emphasized the role of quercetin in BYLY, suggesting that it may be a key molecule in the treatment of RSA. Also, *FOS, HIF1A, IL1B,* and *MYC* might be potential targets. Potential mechanism of quercetin on RSA was further investigated through pharmacological network. Ninety-six core targets were retrieved and the top 20 targets in PPI analysis includes *AKT1, TP53, TNF, IL6, JUN, CASP3, IL1B, EGFR, PTGS2, MYC, HIF1A, MMP9, PPARG, HSP90AA1, CXCL8, CCND1, MMP2, FOS, CCL2,* and *ERBB2* (Supplementary Figures S1A,B). Consistent with the results in BYLY (Figure 3), response to inorganic substance, response to wounding, membrane raft, membrane microdomain, protein domain specific binding, transcription factor binding, kinase binding, and protein kinase binding were also observed in GO enrichment analysis (Supplementary Figure S1C); and NF-kappa B signaling pathway was found in KEGG pathway enrichment analysis (Supplementary Figure S1D). These results indicated the overlapped targets between quercetin and BYLY.

**TABLE 5:** Analysis of core regulatory targets in crucial active components

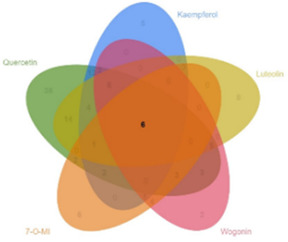 Overlapping	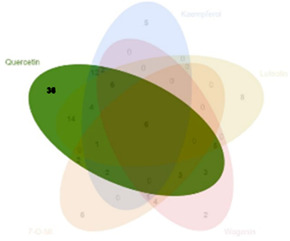 Quercetin specific (Green)				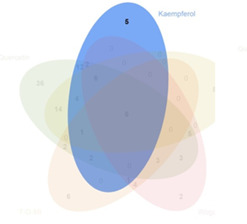 Kaempferol specific (Blue)	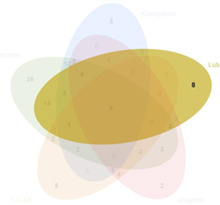 Luteolin specific (Yellow)	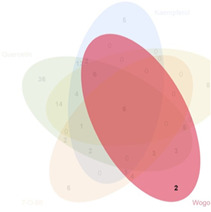 Wogonin specific (Red)	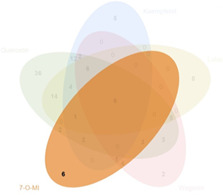 7-O-methylisomucronulatol specific (orange)
*AR*	*ABCG2*	*F3*	*IRF1*	*PON1*	*ALOX5*	*CASP7*	***FN1***	*ADRB1*
*DPP4*	*ACP3*	***FOS***	*MAOB*	*PRKCA*	*MAPK8*	*CDK4*	*KDR*	*CCNA2*
***HSP90AA1***	*CASP8*	*GJA1*	*MMP3*	*RAF1*	*PGR*	*IL4*		*ESR2*
***PPARG***	*CAV1*	***HIF1A***	*MPO*	*RUNX2*	*SLC6A2*	*MDM2*		*HTR2A*
*PTGS1*	*CHEK2*	*HSPB1*	***MYC***	*SERPINE1*	*SLPI*	*MET*		*PIM1*
***PTGS2***	*CRP*	*IGF2*	*ODC1*	*SOD1*		*PCNA*		*SLC6A4*
	*CXCL10*	*IGFBP3*	*PARP1*	*SPP1*		*TYR*		
	*ELK1*	*IL1A*	*PLAT*	*TGFB1*		*XIAP*		
	*ERBB3*	***IL1B***	*PLAU*	*THBD*				

Targets identified as core regulatory targets were underlined and overstruck.

### 3.6 Validation of quercetin in BYLY

**FIGURE 4:**
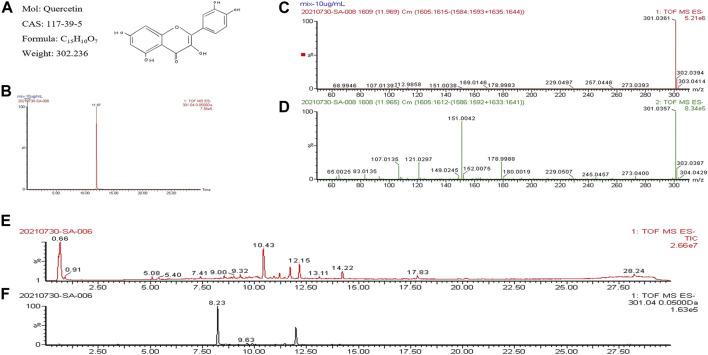
UPLC-Q/TOF-MS analysis of quercetin and BYLY. **(A)** Chemical properties of quercetin. **(B)** Chromatogram of quercetin. **(C)** Low-energy spectrum of quercetin. **(D)** High-energy spectrum of quercetin. **(E)** Total ion chromatogram of BYLY. **(F)** Chromatogram at *m/z* 301.04 of BYLY. Abbreviation: BYLY, *Bushen Yiqi Lixue Yangtai* compounds.

To alleviate the limitations of previous rationalistic work, the UPLC-Q-TOF-MS method was used to confirm quercetin as one of the compounds in BYLY. The chromatographic pattern of quercetin showed that the peak was detected at a retention of time of 11.197 min (Figure 4B). The low-energy spectrum (Figure 4C) showed a peak at *m/z* 301.0361 which corresponded to the molecular weight of quercetin (Figure 4A) and the high-energy spectrum (Figure 4D) showed its fragment ions, further confirming quercetin. Among the 13 peaks in the chromatogram of BYLY (Figure 4E), a peak at 12.15 min was tentatively identified as quercetin. Further analyzing the chromatogram at *m/z* 301.04 (Figure 4F), the peak detected at a retention time of 12.15 min was identified as quercetin. The results validated quercetin as a one of the components in BYLY, indicating its importance in RSA treatment.

### 3.7 Quercetin improved biological function of trophoblast cells under hypoxic conditions

During early pregnancy, extravillous trophoblasts (EVTs) invade the wall of uterine spiral vessels and form temporary plugs, which make the maternal-fetal interface experience a physiological hypoxic condition (Colson et al., 2021; Zhao et al., 2021). Remodeling of the uterine arteries takes place from the fetal side to the maternal side and the uterus becomes nourished by unplugged arteries. Thus, in the first trimester, the villous region suffers lower hypoxic conditions compared with the decidua (Toyooka, 2020; Zhao et al., 2021), indicating the susceptibility of trophoblasts. Under the hypoxia in early pregnancy, hypoxia-inducible factor (HIF) is initiated. As shown in former results, the gene of HIF-1α, *HIF1A*, might take effects (Table 4) in BYLY when treating RSA and the HIF-1α signaling pathway is highly enriched (Figure 3B). According to the PPI analysis (Figure 2D and Supplementary Figure S1B) and GO enrichment analysis (Figure 3A and Supplementary Figure S1D), apoptosis-related molecules (e.g., *CASP3, TNF*), apoptotic signaling pathways and cell proliferative regulation might be involved. Thus, we explored whether quercetin affected the apoptosis or proliferation of a human villous trophoblast cell line, HTR-8/SVneo, under hypoxic conditions.

**FIGURE 5:**
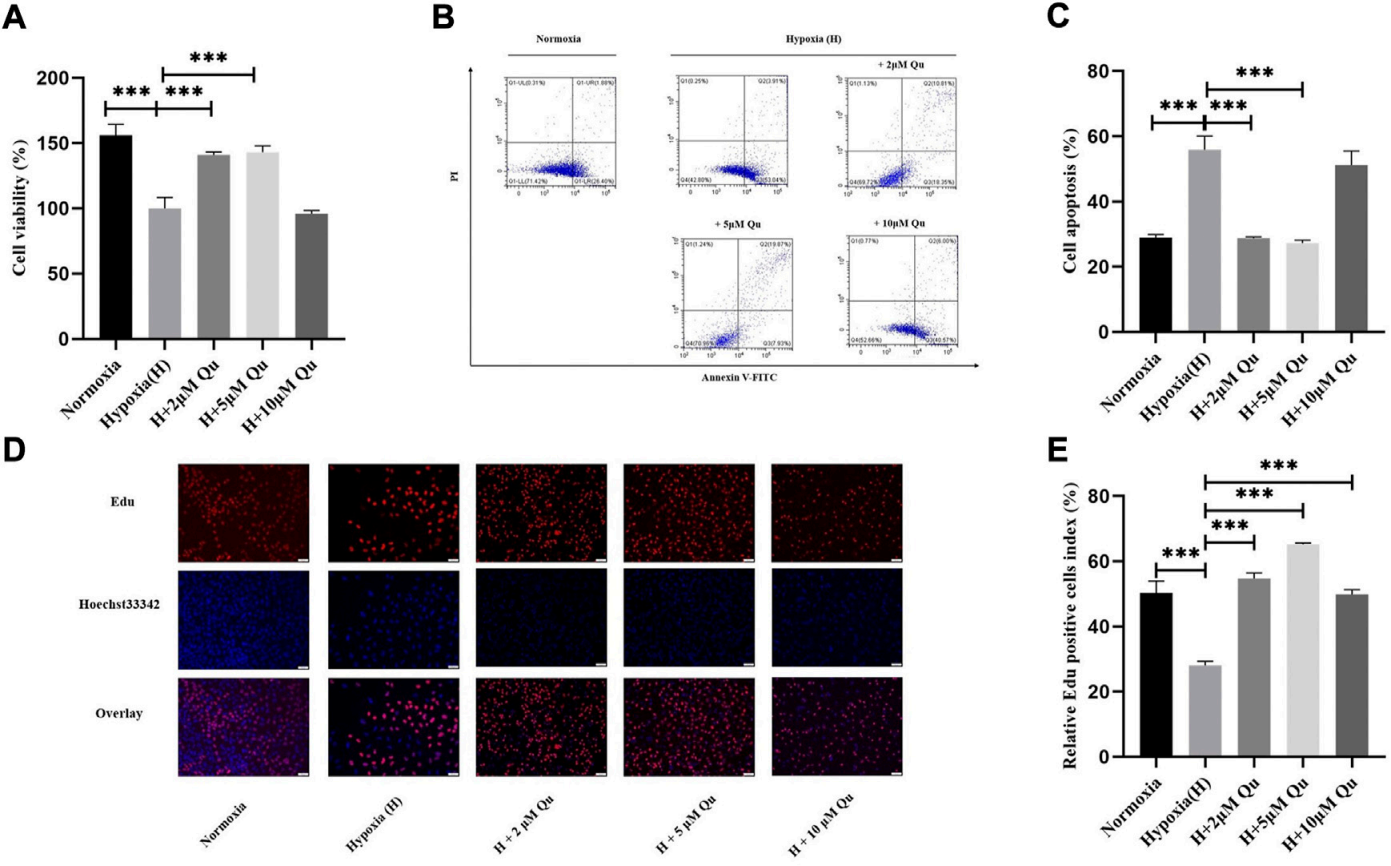
Quercetin improved biological function of trophoblasts under hypoxia condition. **(A)** Cell viability of trophoblasts. **(B)** Flow chart of trophoblasts apoptosis. **(C)** Quantitative analysis of trophoblasts apoptosis. **(D)** Fluorescence images of trophoblasts proliferation. Upper row, EdU fluorescent signals (red); Middle row, Hoechst 33342 fluorescent signals (blue); Lower row, overlapped fluorescent signals. Scale bar: 50 μm. **(E)** Quantitative analysis of trophoblasts proliferation. *n* = 3, ***p < 0.001. Abbreviation: H, hypoxia; Qu, quercetin.

Compared with HTR-8/SVneo in normoxic conditions, cell viability and cell proliferation were significantly decreased (*p* < 0.001), and cell apoptosis was significantly increased (*p* < 0.001) after a 48-h hypoxic incubation. HTR-8/SVneo pretreated with 2 or 5 μM quercetin showed higher cell viability and lower cell apoptosis compared with cells without quercetin treatment under hypoxia (*p* < 0.001) (Figures 5A–C). A concentration of 2/5/10 μM quercetin significantly elevated cell proliferation (*p* < 0.001) compared with hypoxia without quercetin pretreatment, but not in a dose-dependent way (Figures 5D,E). Our results indicated that pretreatment with quercetin in a proper concentration prevented hypoxia-induced cell viability and proliferation reduction as well as cell apoptosis.

### 3.8 Quercetin improved mitochondrial membrane potential and mitochondrial morphology of trophoblast cells under hypoxic conditions

**FIGURE 6:**
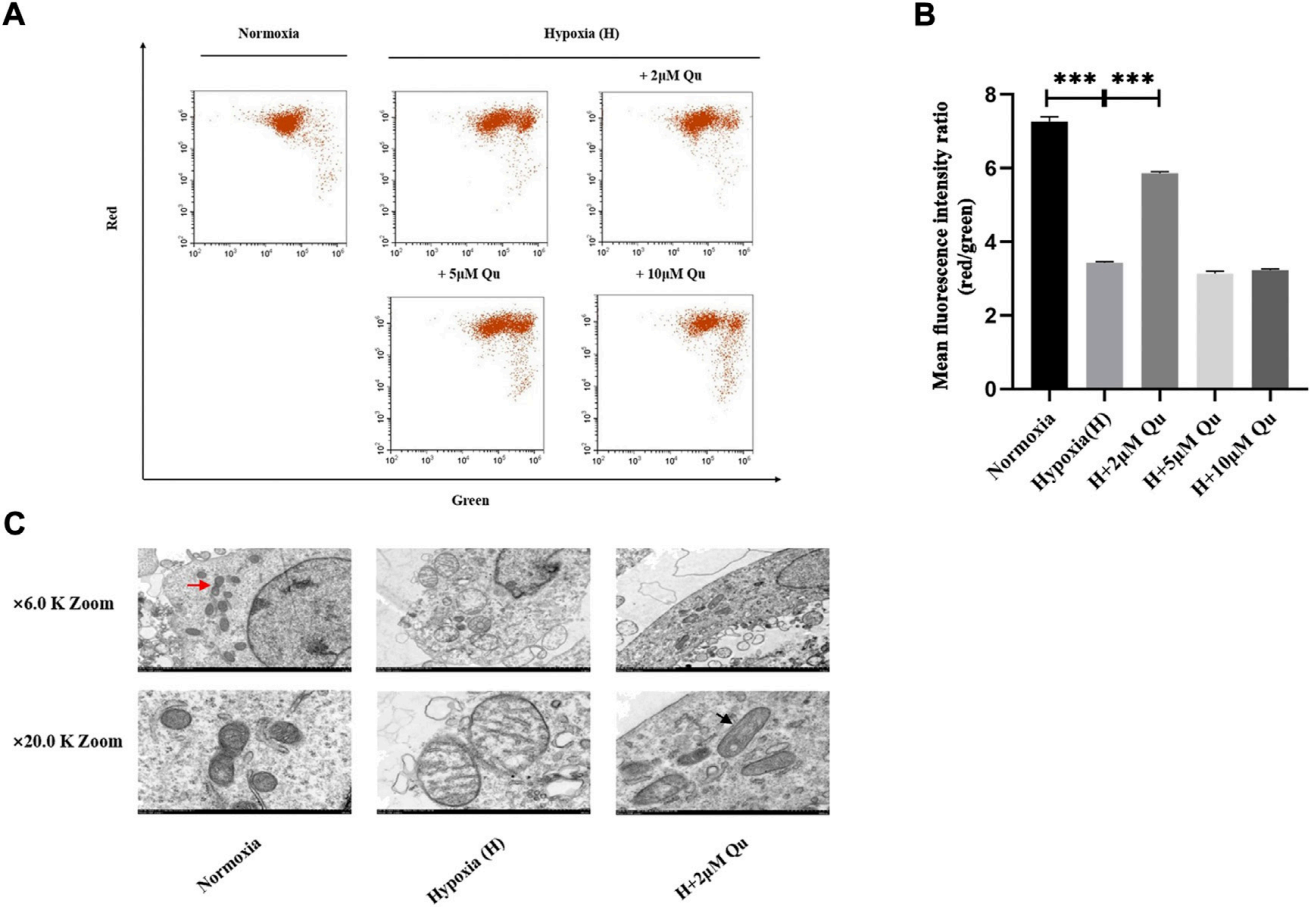
Quercetin improved mitochondrial membrane potential and mitochondrial morphology of trophoblasts under hypoxia condition. **(A)** Flow chart of JC-1 fluorescence intensity. **(B)** Quantitative analysis of JC-1 aggregates (red fluorescence)/monomers (green fluorescence). **(C)** Representative TEM images of mitochondria in HTR-8/Svneo cells. *n* = 3, ***p < 0.001. Abbreviation: TEM, transmission electron microscopy; H, hypoxia; Qu, quercetin.

As shown in Figure 3A and Supplementary Figure S1D, the mitochondrial envelope and mitochondrial outer membrane might be the involved cellular components. Whether quercetin inhibits trophoblast apoptosis through cell-extrinsic or cell-intrinsic processes (mitochondrial-dependent processes) remains unknown. We further investigated the effect of quercetin on trophoblast mitochondrial function. MMP was assessed by JC-1 red/green ratio (Figure 6A and Figure 6B). Red/green ratio was decreased significantly in the hypoxia group compared with the normoxia group (*p* < 0.001). Pretreatment of HTR-8/Svneo cells with 2 μM quercetin could prevent the hypoxia-induced the red/green ratio declination (*p* < 0.001). However, 5/10 μM quercetin pretreatment had no effect on red/green ratio. Thus, a concentration of 2 μM was considered as the effective dose and the ability of 2 μM quercetin pretreatment to prevent mitochondrial morphology change under hypoxia was further studied. TEM pictures of mitochondria are shown in Figure 6C. At low magnification, the mitochondrial density among groups showed no obvious difference, but the shape of mitochondria differed in the hypoxia group and hypoxia with 2 μM quercetin pretreatment group. At high magnification, we could observe mitochondria with normal morphology and clearly identify the cristae and mitochondrial fission/fusion (red arrow). Under the hypoxic environment, irregular mitochondrial swelling and fractured cristae were clearly observed. With 2 μM quercetin pretreatment, the morphological change under hypoxia was improved. Mitochondria appearing as long tubules were observed, which was controlled by mitochondrial fusion (black arrow).

### 3.9 Quercetin inhibited Drp1 expression via increasing miR-34a-5p to suppress mitochondrial fission in trophoblast cells under hypoxic conditions

As shown in our former results, quercetin might regulate mitochondrial function in trophoblasts and mitochondrial dynamic change was observed, which corresponded to the GO enrichment results. Mitochondrial fission and fusion, the equilibrium of which was highly associated with mitochondrial function, were studied to further explore the regulatory role of quercetin. Dynamin-related protein 1 (Drp1) is involved in mitochondrial fission. Mitofusin 1 (Mfn1) and mitofusin 2 (Mfn2) orchestrate the fusion of the outer mitochondrial membrane. Corporately, optic atrophy 1 (Opa1) is responsible for the fusion of the inner mitochondrial membrane (Chan, 2020). In the former GO enrichment analysis, the mitochondrial outer membrane was one of the CP involved in both BYLY and quercetin (Figure 3A and Supplementary Figure S1C). Thus, we further explored the effect of quercetin on mitochondrial dynamics related molecules.

**FIGURE 7:**
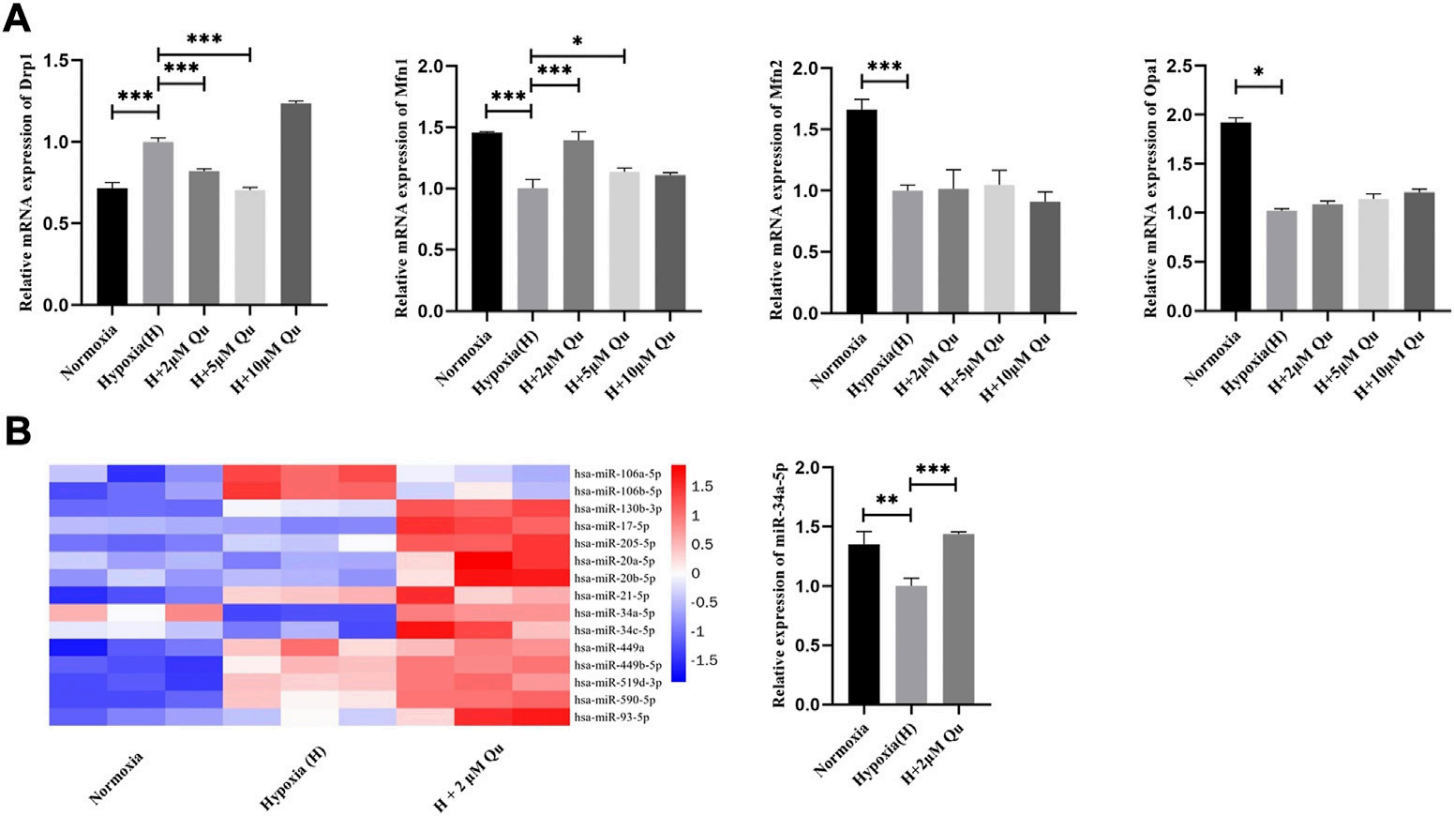
Regulation of quercetin on mitochondrial dynamics and key miRNAs in trophoblasts under hypoxia condition .**(A)** Expression of Drp1, Mfn1, Mfn2 and Opa1 in HTR-8/Svneo cells by qRT-PCR analysis. **(B)** Expression of key miRNAs in HTR-8/SVneo cells by qRT-PCR analysis. *n* = 3, *p < 0.05, ***p < 0.001. Abbreviation: Drp1, dynamin-related protein one; Mfn1, mitofusin one; Mfn2, mitofusin 2; Opa1, optic atrophy 1; H, hypoxia; Qu, quercetin.

Compared with HTR-8/Svneo cells cultured under normoxia, cells under hypoxia showed increased Drp1 mRNA levels (*p* < 0.001) and decreased Mfn1 (*p* < 0.001), Mfn2 (*p* < 0.001), and Opa1 (*p* < 0.05) mRNA levels. Quercetin at 2 and 5 μM concentrations could decrease the mRNA level of Drp1 and increase the mRNA level of Mfn1 compared with the hypoxia group (*p* < 0.001) (Figure 7A). However, quercetin at 2–10 μM concentrations showed no effect on the mRNA expression of Mfn2 and Opa1. These results indicated that quercetin at proper concentrations could prevent Drp1 elevation and Mfn1 declination under hypoxia to different degrees. Since the effect of quercetin on Drp1 was more obvious than it was on Mfn1, we considered Drp1 as the pivotal molecule.

Abundant evidence verified the role of miRNAs as post-transcriptional regulatory factors (Liu et al., 2014). To further explore whether quercetin suppressed Drp1 expression via regulating miRNAs, we forecast possible miRNAs by searching starBase, miRDB, TargetScan, and miRWalk and selected 15 key miRNAs (Supplementary Figure S3). Under normoxia culture and hypoxia culture, with or without quercetin pretreatment, the expression of these 15 miRNAs in HTR-8/SVneo cells were detected by qRT-PCR. As shown in Figure 7B, miR-34a-5p exhibited satisfying expression trends and was significantly suppressed under hypoxia culture compared with the normoxia culture (*p* < 0.01) or with 2 μM quercetin pretreatment (*p* < 0.001).

### 3.10 Quercetin inhibited Drp1 expression in HTR-8/SVneo cells by miR-34a-5p direct regulation

**FIGURE 8:**
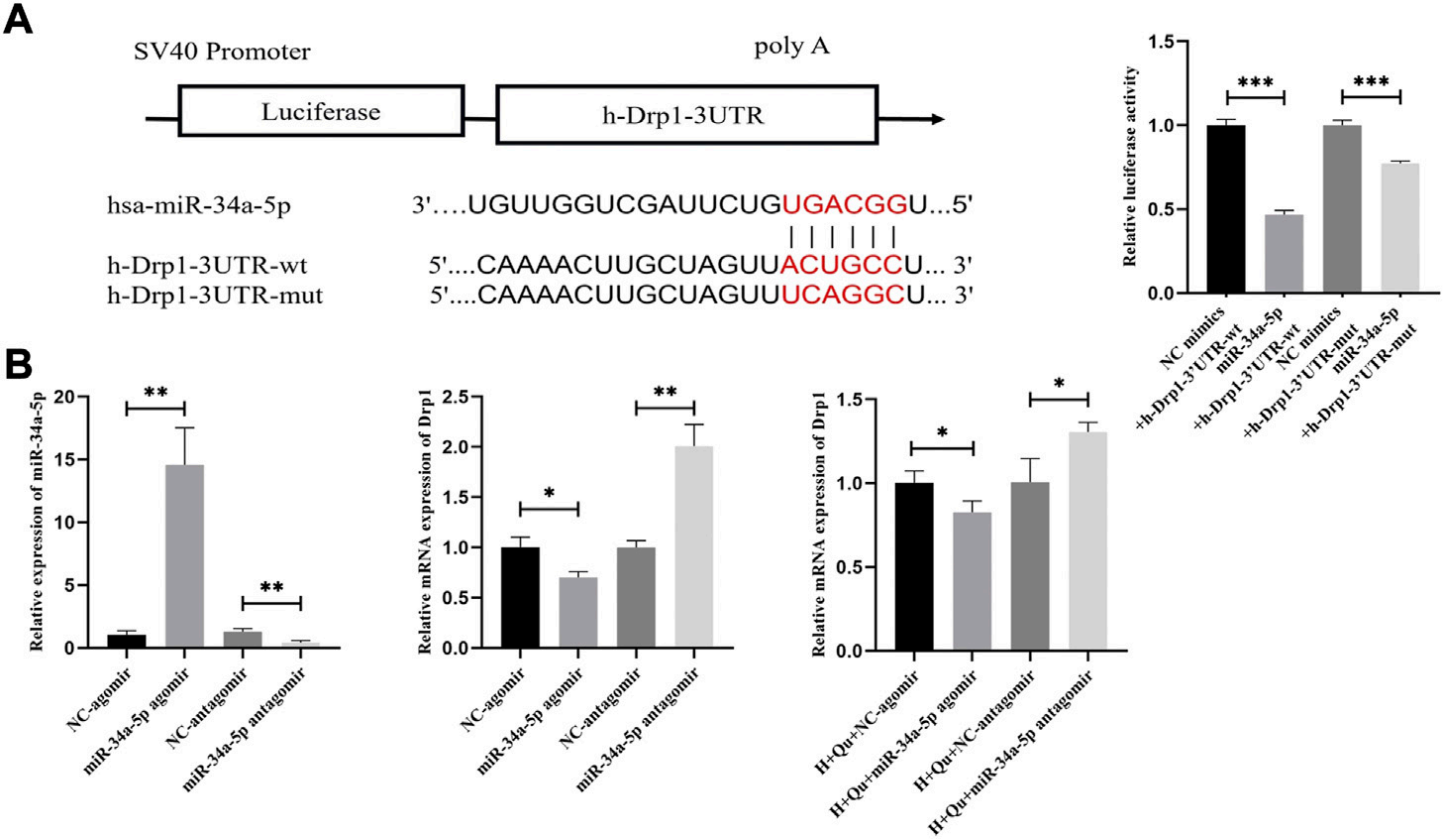
The role of miR-34a-5p in quercetin regulation on Drp1. **(A)** The binding sites between Drp1 and miR-34a-5p; Luciferase activity assays of Drp1-3′UTR-wt and Drp1-3′UTR-mut with or without miR-34a-5p overexpression. **(B)** Expression of miR-34a-5p and Drp1 in HTR-8/SVneo cells by qRT-PCR analysis; Expression of Drp1 in HTR-8/SVneo cells under hypoxia with quercetin pretreatment by qRT-PCR analysis. *n* = 3, *p < 0.05, **p < 0.01, ***p < 0.001. Abbreviation: Drp1, dynamin-related protein one; H, hypoxia; Qu, quercetin; wt, wild-type; mut: mutant; NC: negative control.

We hypothesized that Drp1 was the downstream target of miR-34a-5p in quercetin regulation. The binding sites of miR-34–5p and Drp1 are shown in Figure 8A. To validate the interaction of miR-34a-5 p and Drp1, dual-luciferase report assays were performed by constructing Drp1-3′UTR-wt and Drp1-3′UTR-mut vectors. Overexpression of miR-34a-5p significantly decreased the luciferase activity of Drp1-3′UTR-wt and Drp1-3′UTR-mut (*p* < 0.001) (Figure 8A). However, luciferase activity was suppressed with a higher degree in Drp1-3′UTR-wt groups compared with Drp1-3′UTR-mut. Hence there might be more than one binding site between miR-34a-5 p and Drp1.

To study whether quercetin inhibited Drp1 expression via miR-34a-5p, HTR-8/Svneo cells were transfected with NC-agomir, miR-34a-5p agomir, NC-antagomir, and miR-34a-5p antagomir to overexpress or knockdown miR-34a-5p. The expression of miR34a-5p and Drp1 were studied to validate the regulation of miR34–5p on Drp1 by qRT-PCR (Figure 8B). Moreover, with quercetin pretreatment, the expression of Drp1 was suppressed after overexpressing miR-34a-5p (*p* < 0.05) and promoted after knocking down miR-34a-5p (*p* < 0.05). These results indicated that quercetin inhibited Drp1 expression by regulating miR34-a-5p.

## 4 Discussion

Pregnancy is an event shared by both mothers and fetuses and a disorder of either can induce spontaneous abortion. The concept of BYLY innovatively emphasizes the holistic health of the maternal-fetal organism by combining and balancing *Bushen*, *Yiqi*, *Lixue,* and *Yangtai*. Our preliminary clinical trial validated BYLY as a useful assisting therapy for RSA. The coaction and interaction of various active molecules in BYLY make it difficult to study through *in vitro* research. Thus, highlighted by the study of pharmacological networks, quercetin was considered to be one of the major components for treating RSA and its effect on trophoblasts were further analyzed.

The role of TCM in RSA therapy has been focused on by several trials in recent years. The *Shoutai* Pill is a classic TCM for threating RSA. Li HF *et al.* (Li et al., 2020) reviewed research regarding the *Shoutai* Pill and summarized that its cotreatment with western medicine could prevent miscarriages in the first trimester of pregnancy in women with RSA. Other TCM treatments including the *Gushen Antai* Pill and *Gushen Baotai* decoction have been shown to improve the pregnancy outcomes of RSA (Sun and Zhang, 2019; Lu et al., 2020). However, most of these studies only assessed pregnancy rate but not abortion rate or live birth rate. Furthermore, the insufficient sample size of these studies weakened the supporting argument for using TCM to treat RSA. Although the clinical trials conducted in our study were preliminary and of moderate quality, our results addressed the abortion rate in early pregnancy, and showed a decreased early abortion rate under duphaston combined with BYLY therapy (10.62%), compared with BYLY therapy (29.17%) and duphaston therapy (29.06%). Network pharmacology study was utilized to uncover the potential mechanisms. In PPI network results, *CASP3*, *TNF*, *TP53,* and others indicated the apoptosis or proliferation events, while *MMP9* indicated the migration event. The apoptotic signaling pathway was re-emphasized in GO enrichment analysis. Besides, we noticed the involvement of mitochondria in GO enrichment analysis.

According to network pharmacological results, quercetin is the active component which has most targets. Furthermore, most targets of quercetin (96/141, 68%) are overlapped with the regulatory targets of RSA (Table 3 and Supplementary Figure S1A). Thus, we speculate that quercetin might take greatest effect in BYLY. Quercetin is a secondary metabolite to be found in everyday vegetables and fruits, and this polyphenol has been given much attention in pharmacological research for its accessibility and remarkable efficacy. Its pharmacological properties were elucidated later in the article. Other crucial active components in BYLY, though were not studied in our research, also should be paid attention to. Kaempferol and luteolin, which have similar chemical constructures to quercetin, are flavonoids existed common in nature products. Their molecular mechanisms of action include anticancer, anti-inflammatory, antioxidant, and anti-apoptosis (Imran et al., 2019; Zhu and Xue, 2019; Parent et al., 2020; Jin et al., 2021; Li et al., 2021). Considering their effects during pregnancy, luteolin and kaempferol inhibited infection-induced inflammation in gestational tissues by reducing proinflammatory cytokines (IL-6 and IL-8) and prostaglandins (PGE2 and PGF2α) expression, indicating their potential therapeutic effect for preterm birth (Wall et al., 2013). Besides, luteolin promoted vasorelaxation in uterine arteries during late gestation (Yang D et al., 2021). Since their great pharmacological actions and similar structures to quercetin, the effects of kaempferol and luteolin on maternal-fetal interface are worthy of further exploration. Wogonin is also a kind of flavonoid compound. It has been proved to attenuate liver fibrosis, protect chondrocytes, alleviates hyperglycemia, and protects glomerular podocytes via regulating apoptosis, inflammation, and oxidative stress (Khan et al., 2017; Khan and Kamal, 2019; Dai et al., 2021; Liu et al., 2022). However, few researches investigated the regulation of wogonin during pregnancy. Although no research focuses on the therapeutic effect of 7-O-methylisomucronulatol, it was found to be a key ingredient in several herbs by network pharmacology (Guo et al., 2020; Feng et al., 2021; Zhang and Huang, 2021). Whether it takes effect alone or has synergistic effects with other drugs requires further studies.

In obstetrics and gynecology, the use of quercetin first focused on the treatment of pregnancy-induced hypertension. An important etiology of pregnancy-induced hypertension is oxidative stress which relies on the balance between free radical generation and antioxidant capacity (Ozarowski et al., 2018). As a flavonoid, quercetin is a well-known antioxidant which can eliminate free radical and ROS (Yang D. et al., 2020). Owing to the presence of oxidative stress in embryo development, quercetin was also found to affect pregnancy (Bolouki et al., 2020; Lin and Wang, 2020; Bogacz et al., 2021). Besides its antioxidant effect, growing evidence has uncovered other biological activities of quercetin which shed light on its use in pregnancy loss (Zhou et al., 2020). Combining the results of published papers and our work, regulation of the endocrine-immune network, oxidative stress, and the reproductive endocrine system might play major roles in the effect of quercetin on pregnancy loss therapy. Disorders of the immune microenvironment such as Th1/Th2 and Th17/Treg balance are highly associated with RSA (Qian et al., 2018; Kuroda et al., 2021). The combined use of quercetin and bornyl acetate during the first trimester of pregnancy in mice lowered the CD4^+^/CD8^+^ ratio and IFN-γ/IL-4 ratio, indicating a Th2 shift in the Th1/Th2 balance (Wang et al., 2011). Under an immune-inflammatory response, quercetin protects pregnant mice against adverse effects by repressing IL-6 and IL-8 secretion and upgrading the level of HO-1(Liu et al., 2017). *In vitro* studies on HTR-8/SVneo found that quercetin alleviated oxidative stress by increasing the glutathione (GSH)/oxidized glutathione (GSSG) ratio, reducing caspase3/7 generation and inhibiting JNK/p38 activation (Ebegboni et al., 2019a; Ebegboni et al., 2019b). These results uncovered the anti-inflammatory and anti-oxidative stress effect of quercetin during pregnancy, which agreed with the putative mechanisms in our study. HIF, a heterodimer protein, has alpha (*a*) and beta (*ß*) subunits, and HIF-α contains three isoforms: HIF-1α, HIF-2α, and HIF-3α. Unlike HIF-β, HIF-α is sensitive to hypoxia and is stabilized in the maternal-fetal interface. Our work hypothesized that HIF-1α might be a target of BYLY and showed the protective effect of quercetin on HTR-8/SVneo cells under hypoxia. However, whether quercetin directly targets HIF-1α should be studied in future work. Quercetin is considered to be a phytoestrogen as it can interact with estrogen receptor (ER)-α (D'Arrigo et al., 2021). However, the effect of quercetin on hormone levels is complicated. Quercetin might increase estradiol and progesterone of injured endometrial cells in pregnant rats and exhibit a protective effect (Xu et al., 2014). High dose quercetin treatment on early pregnant rats showed high estradiol levels but low progesterone levels, and these results might be related to the regulation of enzymes that are involved in hormone biosynthesis (Shahzad et al., 2017). Meanwhile, in porcine ovarian granulosa cells, quercetin stimulated progesterone release and had no influence on 17-β estradiol (Kolesarova et al., 2019). The effect of quercetin on reproductive endocrine hormones might differ at different doses and in different tissues.

Mitochondria, the cellular energy centers, are important organelles involved in various biological functions. Pregnancy-relevant events including fertilization, implantation, and fetal development can be affected by mitochondrial function (Benkhalifa et al., 2014). The placenta is a functional organ that forms, develops, and matures throughout pregnancy, which regulates endocrine activities and offers nutrients to fetuses. To coordinate tissue renewal and differentiation, mitochondria are in a balanced state of fission and fusion (Ferree and Shirihai, 2012; Liesa and Shirihai, 2013). Fission machinery is related to Mfn1 and Mfn2 (outer mitochondrial membrane), as well as Opa1 (inner mitochondrial membrane); and fusion machinery is mainly linked with Drp1. Generally, synthesis of new mitochondria (biogenesis) and elimination of damaged mitochondria (mitophagy) relies on mitochondrial fusion and fission, respectively (Ferree and Shirihai, 2012). This quality-control mechanism maintains the health of mitochondria. The balance of mitochondrial fission and fusion has been shown to be important in several complications of pregnancy. Decreased mitochondrial fusion and increased fission have been associated with preeclampsia (Zhou et al., 2017; Ausman et al., 2018; Aydogan Mathyk et al., 2020). Low expression of Mfn2 was related to unexplained RSA (Pang et al., 2013; Cai et al., 2018). However, the role of mitochondrial fission in RSA remained unclear. Since fission and fusion are opposed processes, we speculated that mitochondrial fission might also be important in RSA. Our results uncovered the regulating effect of quercetin on Drp1, while a mild influence was observed.

MiRNAs are a group of small non-coding RNAs, functioning as post-transcriptional regulators by inhibiting transcription or degrading mRNA. Although most miRNAs are in the cytoplasm or nucleus, a small amount of miRNAs are found within mitochondria called mitomiRs (Bandiera et al., 2013). These miRNAs mainly regulate mitochondrial function through affecting the mitochondrial proteome. MitomiRs could affect the tricarboxylic acid cycle, oxidative phosphorylation (OXPHOS), lipid metabolism, mitochondrial dynamics, and mitochondrial-induced apoptosis (Favaro et al., 2010; Yang X. et al., 2020; Agbu and Carthew, 2021; Chen et al., 2021; Ziemann et al., 2022). Drp1 has been shown to be the target of several miRNAs including miR411, miR29a, and miR150–5p. (Jan et al., 2018; Wang Z. J. et al., 2021; Yang W et al., 2021). MiRs-34 belongs to mitomiRs, consistent with the results in our study, but its role in mitochondrial function is not completely understood (Barrey et al., 2011; Geiger and Dalgaard, 2017). Under specific stress, miR-34b/c could control the production of ROS (Pavlides et al., 2010; Consales et al., 2018). MiR-34a are involved in mitochondria-related apoptosis by targeting sirtuin 1 (SIRT1) and decreasing p53 activity (Fang et al., 2017). Regarding the chemotherapeutic effects of quercetin, miRNAs play a pivotal role through its anti-inflammatory, anti-cancer, anti-apoptosis, antioxidant, and metabolism regulation effects (Kim et al., 2019). MiRNA arrays between high quercetin-rich food intake consumers and low quercetin-rich food intake consumers showed significantly different miR-34a expression (*p* < 0.05), indicating the potential regulation of quercetin on miRs-34 (Lam et al., 2012). The results in our work innovatively validated the effect of quercetin on trophoblasts via the miR-34a-5p/Drp1 axis.

The limitations of our study are worthy of remark. The success of BYLY is a combination and balance of nourishing the kidney, reinforcing kidney-*Qi*, promoting circulation, and supporting the fetus. The effect of BYLY relies on the interaction and coaction of various active components. However, our experimental study only observed the effect of quercetin, which is just the tip of the sophisticated network of BYLY. Owing to the difference between *in vivo* and *in vitro* pharmacological activities, the cellular experiments with quercetin cannot fully mimic its effect in the body. Thus, studies on the effect and mechanisms of other pivotal active components and well-designed animal experiments should be emphasized in future work.

## 5 Conclusion

The results in this study showed the clinical efficacy of BYLY, uncovered its potential mechanisms and suggest that quercetin has a role in RSA therapy. Quercetin might protect trophoblasts under hypoxia, manifesting as increased cell viability and proliferation, inhibited apoptosis, and improved mitochondrial function. Quercetin suppression of Drp1 expression via regulating miR-34a-5p might be the underlying mechanisms.

## Data Availability

The original contributions presented in the study are included in the article/Supplementary Material, further inquiries can be directed to the corresponding author.
