# Supplementary material for: The effect of a traditional Chinese quadri-combination therapy and its component quercetin on recurrent spontaneous abortion: A clinical trial, network pharmacology and experiments-based study
Source: Front Pharmacol. 2022 Oct 19;13:965694. doi: 10.3389/fphar.2022.965694 (PMC9626984; doi:10.3389/fphar.2022.965694)
Supplement: Supplementary file 1 [file DataSheet1.PDF]

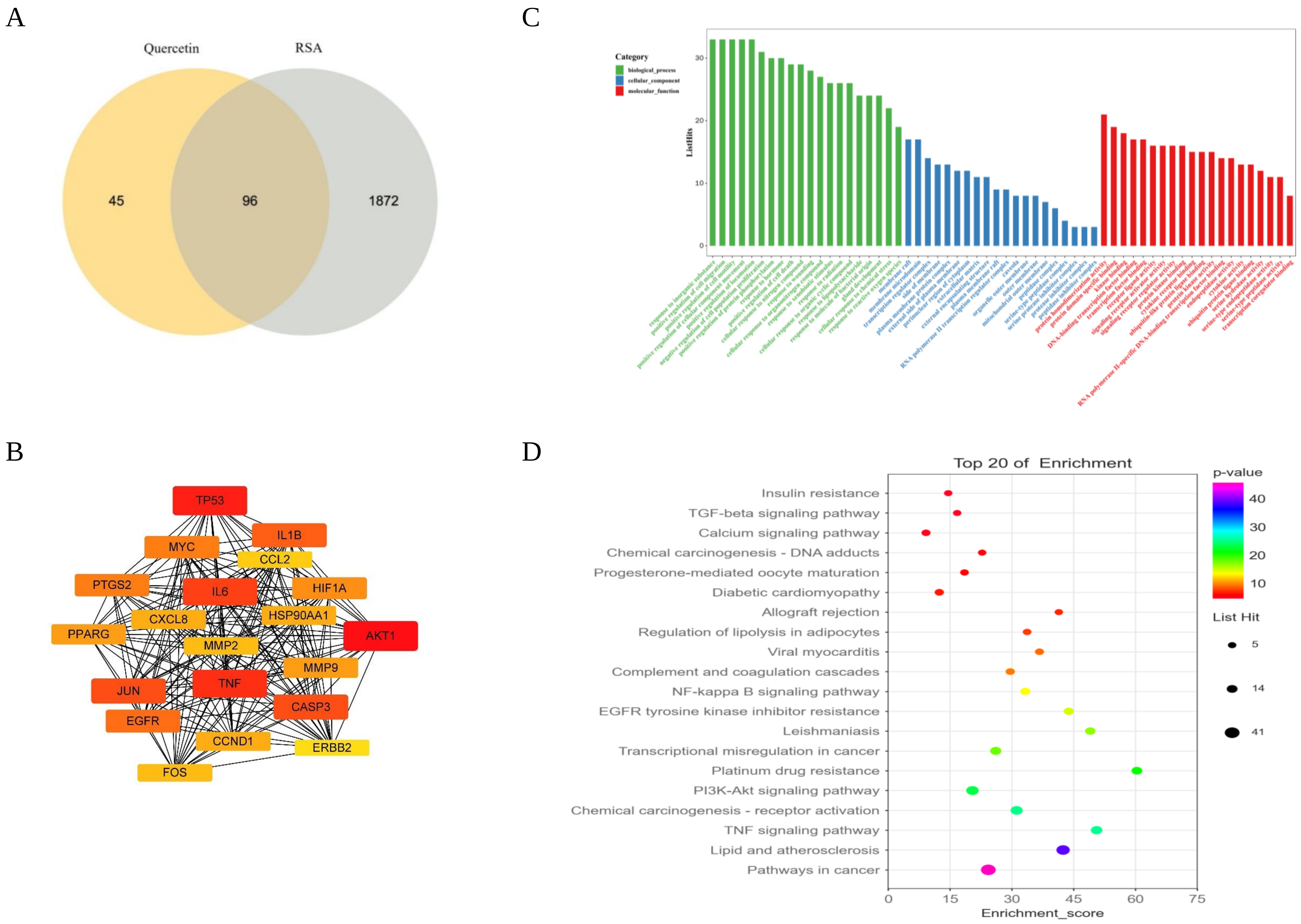

Supplementary Figure S1 | Pharmacological network analysis of quercetin on treating RSA

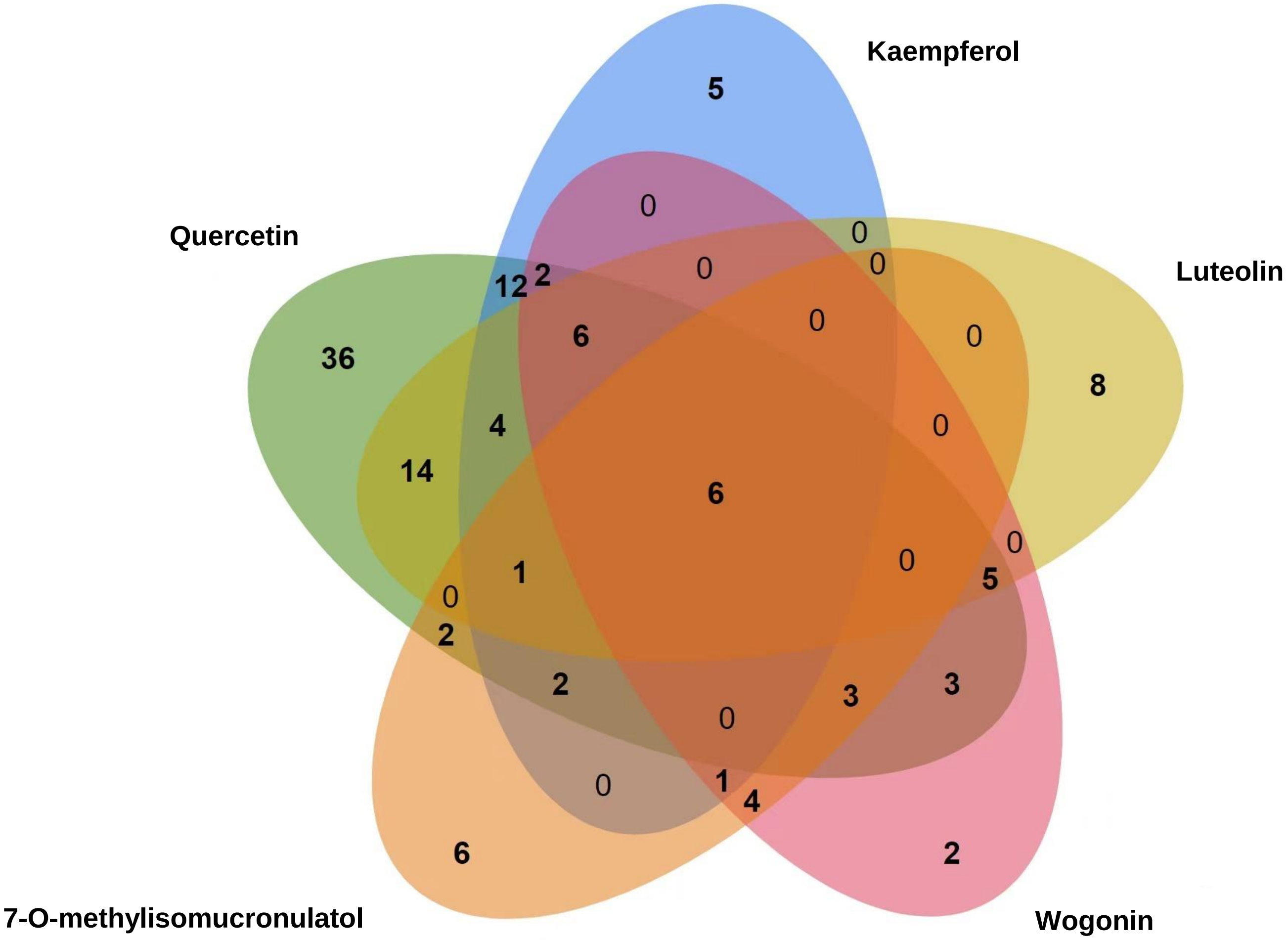

Supplementary Figure S2 | Venn diagram of core regulatory genes in five crucial components in BYLY

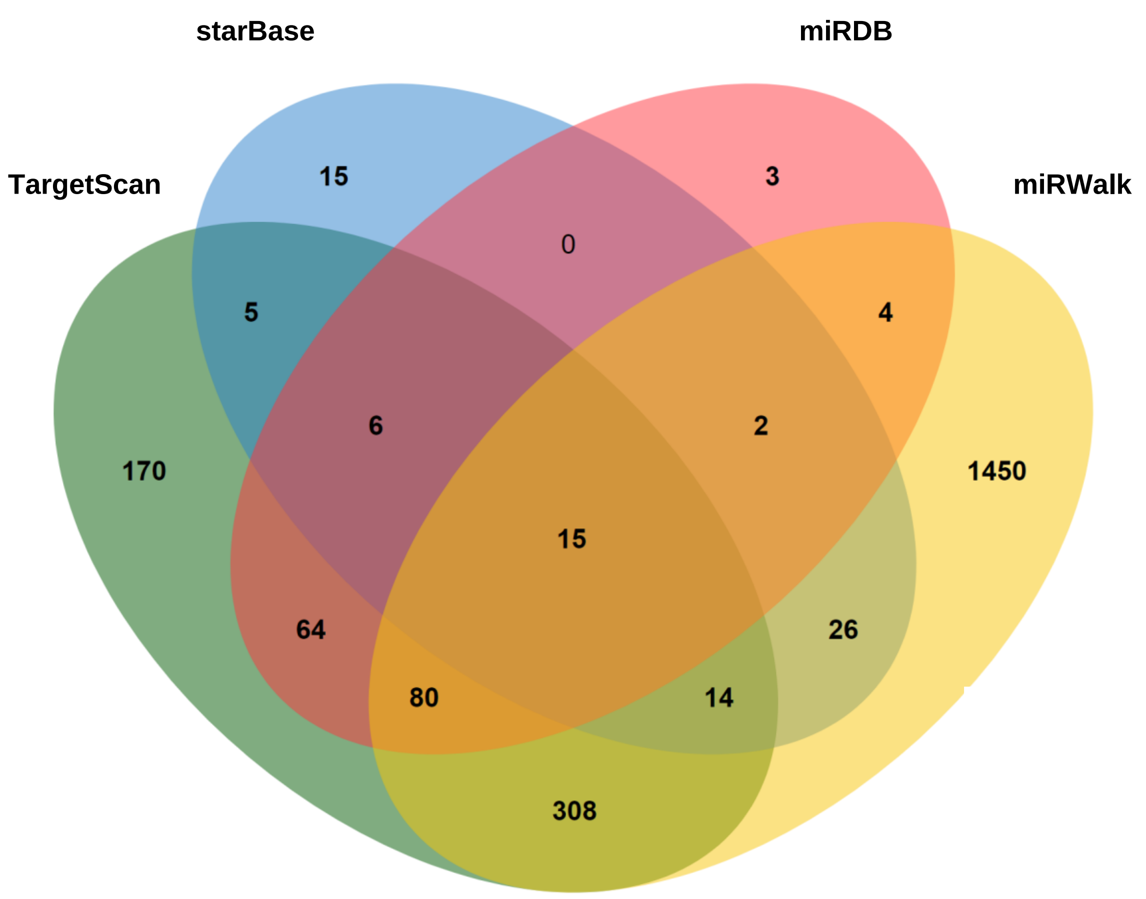

Supplementary Figure S3 | Venn diagram of miRNAs that target Drp1 from four databases
